# Decreased Relative Cerebral Blood Flow in Unmedicated Heroin-Dependent Individuals

**DOI:** 10.3389/fpsyt.2020.00643

**Published:** 2020-07-14

**Authors:** Wenhan Yang, Ru Yang, Fei Tang, Jing Luo, Jun Zhang, Changlong Chen, Chunmei Duan, Yuan Deng, Lidan Fan, Jun Liu

**Affiliations:** ^1^Department of Radiology, Second Xiangya Hospital of Central South University, Changsha, China; ^2^Hunan Judicial Police Vocational College, Changsha, China; ^3^School of Computer Science and Engineering, Central South University, Changsha, China; ^4^Hunan Province Engineering Technology Research Center of Computer Vision and Intelligent Medical Treatment, Changsha, China; ^5^Yunnan Institute for Drug Abuse, Kunming, China

**Keywords:** heroin addiction, magnetic resonance perfusion imaging, arterial spin labeling, neurocognitive, reward circuits

## Abstract

Understanding the brain mechanisms of heroin dependence is invaluable for developing effective treatment. Measurement of regional cerebral blood flow (CBF) provides a method to visualize brain circuits that are functionally impaired by heroin dependence. This study examined regional CBF alterations and their clinical associations in unmedicated heroin-dependent individuals (HDIs) using a relatively large sample. Sixty-eight (42 males, 26 females; age: 40.9 ± 7.3 years) HDIs and forty-seven (34 males, 13 females; age: 39.3 ± 9.2 years) matched healthy controls (HCs) underwent high-resolution T1 and whole-brain arterial spin labeling (ASL) perfusion magnetic resonance imaging (MRI) scans. Additionally, clinical characteristics were collected for neurocognitive assessments. HDIs showed worse neuropsychological performance than HCs and had decreased relative CBF (rCBF) in the bilateral middle frontal gyrus (MFG), inferior temporal gyrus, precuneus, posterior cerebellar lobe, cerebellar vermis, and the midbrain adjacent to the ventral tegmental area; right posterior cingulate gyrus, thalamus, and calcarine. rCBF in the bilateral MFG was negatively correlated with Trail Making Test time in HDIs. HDIs had limbic, frontal, and parietal hypoperfusion areas. Low CBF in the MFG indicated cognitive impairment in HDIs. Together, these findings suggest the MFG as a critical region in HDIs and suggest ASL-derived CBF as a potential marker for use in heroin addiction studies.

## Introduction

Heroin addiction has been a large societal and health problem worldwide for many decades. Long-term use of heroin induces progressive spongiform leukodystrophy, or heroin encephalopathy, resulting in a range of mental symptoms and somatic activity disorders. It is also a major causal factor for the accelerated spread of AIDS, hepatitis C and other major infectious diseases due to the sharing of syringes among patients with addiction (1). While tremendous research advances in heroin addiction have been achieved over the past decades, heroin addiction treatment still has a high relapse rate. A total of 55.8–80% (2, 3) of patients relapsed to heroin use after being abstinent from drug use for 1–3 years at a 12-month follow-up. A major reason for the lack of an effective method for preventing relapse is the lack of a clear picture of heroin addiction-related brain mechanisms (4).

Neuroimaging has been a major tool used to examine brain alterations in heroin addiction/dependence. Brain function involves regional metabolism, which is tightly coupled with cerebral blood flow (CBF) (5). Therefore, measuring regional CBF can enable direct visualization of regional functional brain alterations (6) in heroin-dependent individuals (HDIs). A few studies (7–9) reported decreased CBF in the frontal and parietal lobes in HDIs. While these hypoperfusion patterns are consistent with the frontal and parietal functional impairments often observed in HDIs, relevant findings show large discrepancies across studies, which might be related to the sample sizes included. Some of the hypoperfusion patterns lacked explicit clinical associations. Another major issue is that early studies were based on [18F]-fluoro-deoxy-D-glucose (18F-FDG) positron emission tomography-computed tomography (PET-CT) or single-photon emission computed tomography (SPECT), which require injection of radioactive tracers and are not acceptable to all subjects.

Arterial spin labeling (ASL) perfusion magnetic resonance imaging (MRI) (10, 11) is an MR-based, entirely non-invasive technique for quantifying CBF. ASL uses magnetically labeled arterial blood as an endogenous tracer (12). The perfusion signal is extracted from MR images with labeled arterial blood compared to those without labeling (13). CBF was calculated using the one-compartment model (14) implemented in ASLtbx (15):

(1) $$f=\frac{\Delta M}{2\alpha \cdot M_{0b}\cdot TI_{1}\cdot e^{-TI_{2}/T_{1b}}}$$

where f is the CBF in ml/100 g-min, α is the labeling efficiency (0.9), and TI1 and TI2 are the time of saturation and the time of imaging after applying the spin labeling pulse, respectively. Their values were given above. T1b is the T1 of blood (1.67 s) (16). M0b is the equilibrium magnetization of blood. Because we did not acquire separate M0 images, M0b was approximated by the control image intensity in this study.

CBF measured with ASL MRI has been shown to closely resemble that measured by PET or dynamic susceptibility contrast (DSC) and has high test-retest stability (17, 18). Over time, ASL MRI has been increasingly used in neuropsychiatric studies (19, 20) including drug addiction (9). However, the technique has rarely been used to study heroin addiction.

The purpose of this work was to examine baseline CBF alterations in heroin addiction using ASL perfusion MRI as well as associations of ASL-derived CBF with functional impairments observed in HDIs. Because functional deficits in the prefrontal cortex and the limbic dopamine systems are common in heroin addiction, we hypothesized that HDIs have reduced CBF in those regions, which would be correlated with the behavioral measures of their functional impairments.

## Materials and Methods

### Participant Characteristics

All human studies were approved by the local Institutional Review Board (IRB) of the Second Xiang-Ya Hospital of Central South University. All subjects provided signed written consent forms before participating in any experiments. A total of 72 unmedicated HDIs and 52 demographically matched healthy controls (HCs) were recruited. HDIs were recruited from drug rehabilitation centers in Changsha, Zhuzhou, and Yue Yang (three different cities in Hunan Province), following a positive urine test for heroin, the HDIs underwent compulsory abstinence for no more than 6 months. The HDIs were not taking medications before or during the current study. In total, four HDIs and five HCs were excluded due to poor MR image quality caused by severe head motion and image artifacts. All participants were right-handed Han people. Most participants were smokers, and some also reported alcohol use. The inclusion criteria for the heroin group were as follows: (1) a positive urine test for heroin; (2) a diagnosis of dependence based on criteria outlined in the fourth edition of the Diagnostic and Statistical Manual of Mental Disorders (DSM-IV); (3) a negative urine test for methamphetamine and ketamine; (4) no history of structural brain disease, epilepsy, or head trauma; (5) no contraindications to MRI; and (6) no history of mental or psychiatric illness.

Demographically matched control subjects were recruited through WeChat, flyers, etc., and subjects had to meet the aforementioned inclusion criteria (4), (5), and (6) and have a negative urine test for heroin, methamphetamine, and ketamine. **Table 1** provides the demographic data of the enrolled HDIs and HCs.

**Table 1:** Participant characteristics.

	HDIs (n = 68)(mean ± SD)	HCs (n = 47)(mean ± SD)	t/χ^2^/Z value	*p*
Age (years)	40.9 ± 7.3	39.3 ± 9.2	t = 1.064	0.290
Gender				
Male	42	34		
Female	26	13	χ^2^ = 1.387	.239
Education				
Primary school	3	2	Z = −0.857	.391
Junior high school	50	31		
Senior high school	15	14		
Age of first use (years)	27.69 ± 9.36	–	–	–
Duration of drug use (years)	14.19 ± 7.08	–	–	–
Nicotine use				
Y	62	37		
N	6	10	χ^2^ = 3.598	0.100
Alcohol use				
Y	27	22		
N	41	25	χ^2^ = 0.573	0.450
Handedness	68R	47R	–	–

HDIs, heroin-dependent individuals; HCs, healthy controls.

### Acquisition/Analysis of ASL Data

MRI data acquisitions were conducted in a 3T MRI scanner (MAGNETOM Skyra, Siemens) equipped with a 32-channel receiver coil. Foam padding and a forehead restraining strap were utilized to limit head movement during the scanning procedure. To obtain high-quality ASL data, participants were instructed to remain still with their eyes open during the MRI scan. All participants were allowed a moment to relax and move their hands/feet prior to scanning to ensure the quality of MR images.

T1-weighted high-resolution MRI was acquired using a three-dimensional fast gradient echo sequence with the following parameters: repetition time (TR) = 1450 ms, echo time (TE) = 2.0 ms, inversion time (TI) = 900 ms, field-of-view (FOV) = 256 × 256 mm, slice thickness = 1 mm, and flip angle = 12°. A 5-min 3D ASL scan was performed using a pulsed-ASL sequence from a Siemens device with the following parameters: TR/TE/TI1/TI2 = 2290/16/700/1990 ms, and voxel size = 4 × 4 × 5 mm^3^. Background suppression was applied.

ASL images were preprocessed by ASLtbx (15) using the following steps: motion correction (MoCo) (20, 21), temporal denoising, spatial smoothing, CBF quantification, outlier cleaning, partial volume correction (PVC), spatial registration to the Montreal Neurology Institute (MNI) standard brain space, and CBF extraction for regions of interest (ROIs). Temporal filtering used a high-pass Butterworth filter (cutoff frequency = 0.01 Hz) and temporal nuisance cleaning. Temporal nuisance variables, including the time courses of head motion (three translations and three rotations), the global signal, and the mean CSF signal, were regressed out from the ASL image series at each voxel. The CSF mask was defined during the T1-weighted structural image segmentation. Spatial smoothing was performed with an isotropic Gaussian kernel with a full-width-at-half-maximum (FWHM) of 4 mm. The preprocessed ASL label and control image pairs were then successively subtracted to obtain the perfusion map. The detailed model parameters can be found in previous publications (15, 16). The mean ASL image was registered to the high-resolution structural image. Structural images were segmented into gray matter (GM), white matter (WM), and cerebrospinal fluid (CSF) using the segmentation tool provided in Statistical Parametric Mapping (https://www.fil.ion.ucl.ac.uk/spm/software/spm12/), projected into the ASL image space based on the registration transform between the mean ASL control image and the structural image and subsequently used to extract the CBF signals for temporal denoising and PVC. The relative CBF (rCBF) map (22, 23) was calculated by dividing the perfusion maps by the global mean perfusion value from the GM and WM.

**Figure 1:**
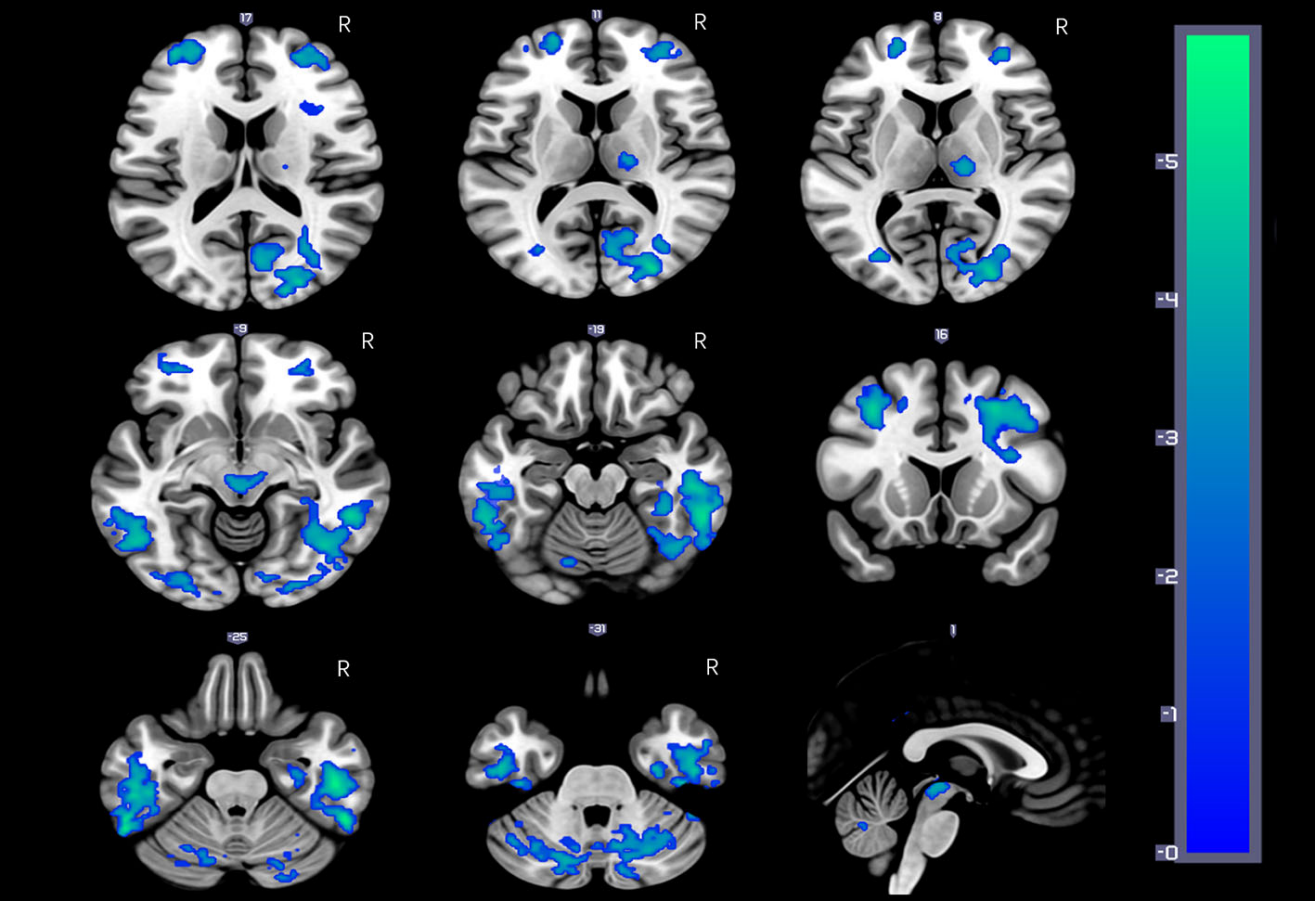
HDIs show significantly reduced rCBF in the bilateral inferior temporal gyrus (ITG), middle frontal gyrus (MFG), cerebellar vermis, and posterior cerebellar lobe, precuneus; the right posterior cingulate gyrus (PCG) and thalamus; and the midbrain adjacent to the ventral tegmental area (VTA) (FDR correction, q = 0.05, p = 0.00158, t = 3.012).

Voxel-wise CBF diﬀerences between the two groups were computed using two-sample t-tests, the result was showed in **Figure 1**, age, gender, education, smoking, and drinking as covariates. Diﬀerences in the rCBF from group comparisons were corrected for multiple comparisons to a signiﬁcance level of p < 0.05 by false discovery rate (FDR) correction. The ROIs from whole-brain voxel-wise comparisons (**Figure 1**) mainly include nine cerebral regions according to the automated anatomical labeling (AAL) template: the bilateral middle frontal gyrus (MFG), inferior temporal gyrus (ITG), and precuneus (PCUN); the right posterior cingulate gyrus (PCG), calcarine area (CAL), and thalamus. Then, nine sub-ROIs were generated from the intersection of voxel-wise ROIs and each regional AAL template, and each sub-ROI of rCBF values was calculated for the two groups. The rCBF values extracted by each sub-ROI between the two groups were compared using two-sample t-tests (**Table 3**) and evaluated for possible associations with neuropsychological characteristics.

### Clinical Characteristics and Neurocognitive Measures

The duration of heroin use, the dose consumed, and other clinical characteristics was also collected from the HDIs. Cognitive function tests, including the Wechsler Adult Intelligence Scale Third Edition (WAIS-3) Digit Symbol Test (including the first and second minutes), forward and backward digit memory span tasks, and Trail Making Test (TMT) were completed by all patients following MRI examination. The Digit Symbol Test score is a part of the WAIS and an indicator of the executive function of the frontal lobe based on visual, spatial and motor processing speed. Participants were asked to write as many numbers as possible that were paired with symbols within 2 min (24–26). The forward and backward digit memory span tasks have been widely used for simple clinical measurements of working memory. The digit span task required the subjects to remember the numbers on an answer sheet in both forward and backward order. This test involves working memory, which can reflect particular aspects of the cognitive function of the brain (27–29). The TMT (30) required the subjects to connect scattered numbers in the correct sequence on a sheet of paper quickly and accurately. They could not skip numbers. The TMT was used to evaluate psychomotor performance (31).

### Statistical Analysis

Statistical analyses were performed using SPSS 18.0. The HDI and HC groups were compared using a two-sample t-test for age, the chi-square test for smoking and drinking and rank sum tests for education levels, and the signiﬁcance level was set to p < 0.05. The data distributions were tested for normality using the Kolmogorov-Smirnov test. Normally distributed data were analyzed using independent-samples t-tests, while non-normally distributed data were analyzed using rank sum tests.

Voxel-wise CBF diﬀerences between the two groups were computed using two-sample t-tests. Diﬀerences in rCBF from group comparisons were corrected for multiple comparisons to a signiﬁcance level of p < 0.05 by FDR correction [q = 0.05, p = 0.00158, t (113) = −3.012] and a cluster size threshold of 100. Correlations between rCBF levels and clinical characteristics were evaluated using Pearson’s correlation coefficients. The level of statistical significance was set at p < 0.05.

## Results

### ASL Results

**Figure 1** and **Table 2** show the whole-brain cross-sectional rCBF comparison results. The statistical threshold of p = 0.00158 was corrected for multiple comparisons with False FDR correction. Compared to HCs, HDIs showed significant rCBF decreases in the bilateral cortical and subcortical ITG, MFG, cerebellar vermis, posterior cerebellar lobe, and PCUN; the right PCG and thalamus; and the midbrain adjacent to the ventral tegmental area (VTA) (**Figure 1**, **Table 2**). Many significant differences were shown in the rCBF values extracted from overlapping ROI masks, and 3 rCBF values were excluded due to negative values (1 in the bilateral PCUN and 1 in the right thalamus) (**Table 3**). No increased regional rCBF areas were detected in HDIs compared with the control group (FDR correction).

**Table 2:** Brain areas with significantly lower rCBF in HDIs.

Brain areas (AAL)	R/L	MNI coordinates			Voxels	Peak intensity
		x	y	z		
Middle frontal gyrus /medial frontal gyrus/OMFC	R	26	12	46	2450	−5.1791
Middle frontal gyrus /medial frontal gyrus/OMFC	L	−34	18	46	644	−5.5855
Inferior temporal gyrus	R	50	−22	−24	2081	−5.7475
Inferior temporal gyrus /fusiform gyrus	L	−56	−52	−24	1047	−5.5997
Posterior cingulate cortex/calcarine	R	16	−66	12	161	−3.9249
Cerebellar posterior lobe /cerebellar declive	L	−16	−76	−28	262	−4.2837
Thalamus	R	6	−22	−4	260	−3.9918
Cerebellar posterior lobe /cerebellar declive	R	26	−68	−30	150	−3.7962
Precuneus	R	28	−62	−38	220	−4.3007
Precuneus	L	−10	−54	44	414	−4.4647
Midbrain (VTA)/right thalamus	B/R	0	−28	−8	118	−3.9221

The statistical threshold of p = 0.00158 was corrected for multiple comparisons with false discovery rate (FDR) correction, and the cluster size = 100; coordinates are located in Montreal Neurological Institute (MNI) space; OMFC, orbital medial frontal cortex; VTA, ventral tegmental area; B, bilateral; AAL, automated anatomical labeling template.

**Table 3:** rCBF of ROI mask.

Brain areas	Patients(mean ± SD)	Controls(mean ± SD)	*t*	*p*
MFG.L	1.28 ± 0.40	1.69 ± 0.27	t (113) = −6.66	.000
MFG.R	1.35 ± 0.41	1.72 ± 0.25	t (113) = −6.11	.000
ITG.L	1.15 ± 0.39	1.51 ± 0.32	t (113) = −5.37	.000
ITG.R	1.22 ± 0.36	1.58 ± 0.28	t (113) = −5.92	.000
PCUN.L	1.60 ± 0.44	1.96 ± 0.31	t (112) = −5.27	.000
PCUN.R	1.78 ± 0.48	2.15 ± 0.37	t (112) = −4.42	.000
THA.R	1.41 ± 0.47	1.68 ± 0.33	t (113) = −3.68	.000
PCG.R	1.09 ± 0.32	1.27 ± 0.31	t (112) = −3.05	.003
CAL.R	1.74 ± 0.64	2.18 ± 0.40	t (113) = −4.48	.000

MFG.L, left middle frontal gyrus (47 HCs + 68 HDIs); MFG.R, right middle frontal gyrus (47 HCs + 68 HDIs); ITG.L, left inferior temporal gyrus (47 HCs + 68 HDIs); ITG.R, right inferior temporal gyrus (47 HCs + 68 HDIs); PCUN.L, left precuneus (46 HCs + 68 HDIs); PCUN.R, right precuneus (46 HCs + 68 HDIs); THA.R, right thalamus (47 HCs + 68 HDIs); PCG.R, right posterior cingulate gyrus (46 HCs + 68 HDIs); CAL.R, right calcarine (47 HCs + 68 HDIs).

### Neuropsychological Tests

Compared to HCs, HDIs showed significant differences in the WAIS-R Digit Symbol Test 2nd-minute scores [*t*_(73)_ = −2.663, p = 0.011], backward digit memory span [*t*_(70)_ = −2.557, p = 0.013], and TMT times [*t*_(76)_ = 3.556, p = 0.001]. No significant differences were observed between forward digit memory span [*t*_(70)_ = −1.730, p = 0.086] and Digit Symbol Test 1st-minute scores [*t*_(73)_ = −1.689, p = 0.095].

### Correlations Between CBF and Neuropsychological Test Performance

**Figure 2:**
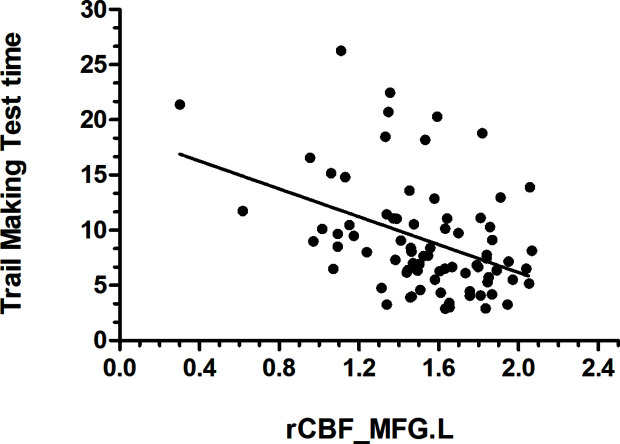
rCBF value in MFG.L was negatively correlated with Trail Making Test completion time (s) (r = −0.413, p < 0.001; N=36 HDIs + 42 HCs); MFG. L, left middle frontal gyrus.

**Figure 3:**
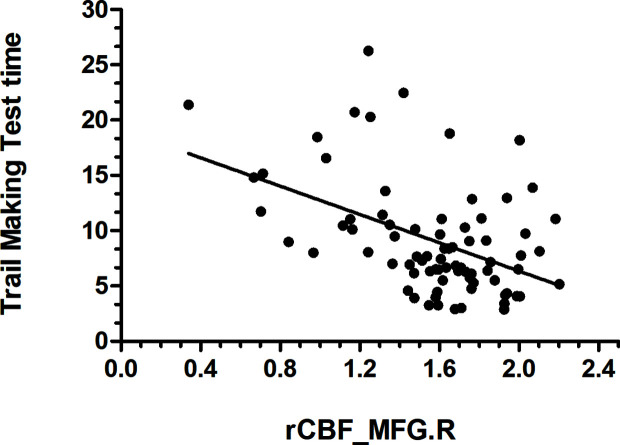
rCBF value in MFG.R was negatively correlatedwith Trail Making Test Time completion time (r = −0.466, p < 0.001; N=36 HDIs + 42 HCs); MFG.R, right middle frontal gyrus.

Data from the neuropsychological tests completed by 36 patients and 42 HCs were used to assess the significance of correlations between test performance and rCBF. rCBF in the left MFG and right MFG was negatively correlated with the TMT times (left: r = −0.413, p < 0.001; right: r = −0.466, p < 0.001) (**Figures 2** and **3**). No significant correlation was observed between rCBF in the bilateral MFG/ITG and the other neuropsychological test results.

## Discussion

Using ASL perfusion MRI, we identified hypoperfusion in frontal, temporal, and parietal areas in HDIs, which was correlated with neuropsychological impairments in the patients.

Hypoperfusion in HDIs has been reported in several studies. Denier et al. (9) found hypoperfusion in frontal and temporal areas following acute administration of heroin to HDIs. Using SPECT, Gilberto et al. (7) demonstrated reduced CBF in the right frontal and left temporal lobes in opioid-dependent subjects with comorbid depression. Lukas et al. (8) investigated opioid-dependent subjects with SPECT and reported decreased CBF in the bilateral cortical prefrontal lobe, right thalamus and hippocampus in opioid-dependent patients. While our hypoperfusion findings in HDIs were partially consistent with those of previous studies, we used ASL MRI, which is non-invasive and does not require radioactive materials. Another merit of our study was the relatively large sample size.

Mesolimbic system dysfunction is a hallmark change in drug addiction (32, 33), including heroin addiction (34–36). By binding to the µ-opioid receptors on GABA neurons in the midbrain, heroin suppresses the inhibitory effects of GABA on dopamine neurons, which subsequently promotes the release of dopamine, leading to elevated reward effects (37). Reduced rCBF in mesolimbic areas in HDIs may be a result of that hallmark change and is consistent with various dysfunctions (such as increased impulsivity and altered reward processing) involving those regions as identified by previous neuroimaging and neurobiological studies (33).

Frontal brain alterations represent a second hallmark symptom of drug addiction, which is often characterized by a loss of executive function, especially inhibition (38). Our frontal hypoperfusion findings in HDIs align with that symptom, which also establishes ASL MRI as a useful tool for measuring such frontal brain dysfunction in drug addiction. The clinical relevance of the frontal hypoperfusion patterns was further demonstrated by the rCBF vs TMT correlations. The TMT reflects the capabilities of mental flexibility, working memory, and executive function (38, 39). The negative frontal rCBF vs TMT processing time in HDIs indicates that patients with lower rCBF in the frontal cortex have more-impaired executive functions.

We found significantly reduced rCBF in the precuneus and ITG in HDIs. The precuneus and ITG are part of the default mode network (DMN). Hypoperfusion in the ITG and precuneus might be a result of altered facilitation function of the DMN, which has been shown to be affected by many neuropsychiatric diseases (40, 41). Reduced rCBF in the precuneus is consistent with the reduced structural and functional connectivity of the precuneus in HDIs, as reported in (42–44), and may reflect functional alterations regarding memory consolidation and information processing, especially in relation to drug cues (45–47). The ITG plays an important role in the connection of visual, linguistic and multisensory processing (47, 48). However, the role of the ITG in heroin addiction remains unclear. Our finding of reduced ITG rCBF is consistent with the hypoperfusion in the bilateral temporal gyrus as measured with SPECT (single photon emission computed tomography) (49).

Decreased CBF was detected in the cerebellar vermis and cerebellar posterior lobe. Studies have shown that the vermis is involved in the regulation of both emotional and cognitive processes (49–52). Thus, further research is needed to clarify the relationship between alterations in CBF and cognitive function in HDIs.

Using ASL, we demonstrated that frontal cognitive impairment is a major element of dysfunction in abstinent HDIs. The limitation of this study is that it had a cross-sectional design. Further studies are required to confirm the relationship between alterations in brain CBF and impaired cognitive function after abstinence.

## Conclusions

In summary, using a relatively large sample, we identified hypoperfusion patterns in mesolimbic, frontal, parietal, and temporal regions in HDIs. Hypoperfusion in frontal brain regions might be a primary marker for heroin addiction as supported by the cross-sectional differences and correlations with cognitive impairment identified.

## Data Availability Statement

All datasets generated for this study are included in the article/supplementary material.

## Ethics Statement

The studies involving human participants were reviewed and approved by Institutional Review Board of the Second Xiang-Ya Hospital of Central South University. The patients/participants provided their written informed consent to participate in this study. Written informed consent was obtained from the individual(s) for the publication of any potentially identifiable images or data included in this article.

## Author Contributions

WY, JiL, JZ, and JuL conceptualized and designed the study. WY, JiL, FT, and LF conducted the behavioral and imaging analyses. RY, CC, CD, and YD conducted the assessments. JuL modiﬁed the manuscript and supervised the study. WY wrote the ﬁrst draft and all authors provided input on the ﬁnal version of the manuscript.

## Funding

The National Key Research and Development Program of China (Grant number: 2016YFC0800908) and the National Natural Science Foundation of China (U1052225). National Natural Science Foundation of China, Grant Number: 61971451; Innovative Province special construction foundation of Hunan Province, China, Grant Number: 2019SK2131.

## Conflict of Interest

The authors declare that the research was conducted in the absence of any commercial or financial relationships that could be construed as a potential conflict of interest.
